# Latency, Integration, and Reactivation of Human Herpesvirus-6

**DOI:** 10.3390/v9070194

**Published:** 2017-07-24

**Authors:** Shara N. Pantry, Peter G. Medveczky

**Affiliations:** 1 College of Medicine, University of South Florida, 12901 Bruce B. Downs Blvd, MDC Box 7, Tampa, FL 33612, USA; spantry@health.usf.edu; 2 Miller School of Medicine, University of Miami, Life Sciences and Technology Park, 1951 NW 7th Avenue Ste. 270, Miami, FL 33136, USA

**Keywords:** HHV-6, integration, telomere, latency, inherited herpesvirus, human herpesvirus-6, immune tolerance, superinfection

## Abstract

Human herpesvirus-6A (HHV-6A) and human herpesvirus-6B (HHV-6B) are two closely related viruses that infect T-cells. Both HHV-6A and HHV-6B possess telomere-like repeats at the terminal regions of their genomes that facilitate latency by integration into the host telomeres, rather than by episome formation. In about 1% of the human population, human herpes virus-6 (HHV-6) integration into germline cells allows the viral genome to be passed down from one generation to the other; this condition is called inherited chromosomally integrated HHV-6 (iciHHV-6). This review will cover the history of HHV-6 and recent works that define the biological differences between HHV-6A and HHV-6B. Additionally, HHV-6 integration and inheritance, the capacity for reactivation and superinfection of iciHHV-6 individuals with a second strain of HHV-6, and the role of hypomethylation of human chromosomes during integration are discussed. Overall, the data suggest that integration of HHV-6 in telomeres represent a unique mechanism of viral latency and offers a novel tool to study not only HHV-6 pathogenesis, but also telomere biology. Paradoxically, the integrated viral genome is often defective especially as seen in iciHHV-6 harboring individuals. Finally, gaps in the field of HHV-6 research are presented and future studies are proposed.

## 1. The Discovery of HHV-6

Human herpesvirus-6 (HHV-6), originally designated human B-lymphotropic virus, was isolated from patients with acquired immunodeficiency syndrome (AIDS)-related lymphomas, lymphoadenopathies, and other lymphoproliferative disorders in North America in 1986 [1]. Initial studies identified the virus as a novel herpesvirus that primarily infects B-lymphocytes. However, later studies demonstrated that HHV-6 selectively infects and replicates in T-lymphocytes with a preference towards cluster of differentiation 4-positive (CD4+) T-cells [1,2,3,4]. After the initial discovery of HHV-6 and the isolation of several other strains, it became apparent that the HHV-6 viruses could be separated into two distinct variants, HHV-6A and HHV-6B. This separation was based on distinct antibody reactivity, T-cell clone reactivity, and restriction banding patterns [5,6,7]. More recently, the International Committee on Taxonomy of Viruses (ICTV) has recognized the HHV-6 variants as two distinct viruses [8]. The basis for separation comes in the form of epidemiological data as well as biological and immunologic properties.

## 2. Biological Differences between HHV-6A and HHV-6B 

Human herpesvirus-6A (HHV-6A) and human herpesvirus-6B (HHV-6B) both replicate in T-cells, however, the viruses differ in receptor usage. It is well agreed upon that the primary receptor for HHV-6A viruses is the human cluster of differenction 46 (CD46) receptor, which is found on all nucleated human cells (reviewed in [9]). Conversely, utilization of the CD46 receptor by HHV-6B viruses depends greatly on the virus strain as well as the phenotype of the cell line being infected. Recent studies indicate that the primary receptor for HHV-6B is cluster of differentiation 134 (CD134), a member of the tumor necrosis factor (TNF) superfamily that is present on activated T-cells [10]. Transduction of HHV-6B non-permissive cell lines, JJHan or SupT1, with CD134 allowed infection by HHV-6B HST or KYO [10,11]. Furthermore, the HHV-6B Z29 gH/gL/gQ1/gQ2 glycoprotein complex is incapable of binding to CD46 and causing productive infection in CD134− cell lines [12]. While the studies identifying CD134 as the HHV-6B receptor used strains HST, Z29, and KYO, another study utilizing HHV-6B strain PL1 reported that infectivity greatly depended on the CD46 isoform expression of the target cell. HHV-6B PL1 successfully infected the CD134 Molt3 and SupT1 cell lines by use of the CD46 C1 and C2 isoforms [13]. Sequence analyses of clinical samples indicate that HHV-6B viruses can be segregated into two subgroups based on differences in the envelope glycoprotein H (gH) amino acid sequence [14]. Therefore, it is possible that differences in receptor-binding glycoprotein sequences between lab-adapted strains could account for the aforementioned differences in HHV-6B receptor usage.

## 3. Epidemiology of HHV-6

The seroprevalence of the HHV-6 viruses ranges up to 100% in most regions of the world [15]. In the Lusaka, Zambia region of Sub-Saharan Africa, HHV-6A prevalence was initially reported to be 85% in healthy children and 57% in symptomatic children aged six or eight years old [16]. However, a more recent, larger-scale study conducted in the same region of Zambia reported an HHV-6A prevalence of less than 1% and an HHV-6B prevalence of approximately 20% in hospitalized children under the age of two [17]. In the United States, Japan, and regions of Europe the HHV-6B is the predominant cause of childhood HHV-6 infections [18,19]. In these regions, primary HHV-6B infection typically occurs by the age of two, and is the etiological cause of exanthema subitum, also known as roseola infantum [20]. Another closely related virus, human herpesvirus-7 (HHV-7) is also recognized as a causative agent of exanthema subitum [21,22] Nevertheless, specific diseases associated with HHV-6A have yet to be identified. HHV-6 infection has also been associated with multiple sclerosis [23,24,25] and chronic fatigue syndrome [26,27,28], but further studies are needed to establish causation between HHV-6 infection and the aforementioned disease states. 

## 4. HHV-6 Genome Structure

**Figure 1:**
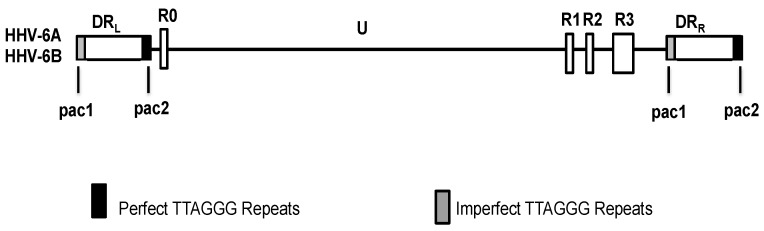
Genome organization of human herpesvirus-6 (HHV-6). Solid lines represent the unique length (U) regions of the genome, boxed regions represent repetitive elements of the genome, and the black boxes represent the perfect telomere repeats, while the gray boxes represent the imperfect telomere repeats. DR_L_: Direct repeat left; DR_R_: Direct repeat right; pac1, pac2: Packaging signals; R0–3: Repetitive elements.

The genome size of HHV-6A or HHV-6B viruses ranges from 159 to 162 kbp and consists of several distinct regions (Figure 1) [29,30,31]. The central unique region encodes the majority of the protein-coding open reading frames. Two blocks of 8–13 kb direct repeats, designated direct repeat left (DR_L_) or right (DR_R_), flank each side of the unique region; on either side of the DR_R_ and DR_L_ are pac1 and pac2 packaging signals [29,30,31,32]. Adjacent to these packaging signals are four regions that contain telomere-like sequences. The HHV-6 hexanucleotide telomere sequences (TTAGGG)_n_ are identical to the human telomere sequence, and may occur as perfect telomere repeats in tandem or as imperfect repeats that are interrupted by a variable number of other nucleotides [30]. On average, there are approximately 50–60 telomeric repeats at the left end of the viral genome [31,32,33]. The genomes of HHV-6A and HHV-6B also contain major repetitive elements located near the right end of the unique length region: R1, R2, and R3, and HHV-6B has an additional repetitive element R0 located at the junction of the DR_L_ and the unique segment [29,31].

## 5. Integration of HHV-6 in Telomeres during Latency

Like all herpesviruses, HHV-6 establishes lifelong latency in which the viral genome is maintained and distributed to daughter cells without infectious virus production and viral gene expression is limited. The proposed site of HHV-6 latency is the monocyte/macrophage cell population [34]. However, during in vitro infection latency has also been established for both HHV-6A and HHV-6B in bone marrow progenitor cells [35] and T-cells [36,37]. Additionally, in vitro latency of HHV-6B has been established in astrocytes [38], while latency of HHV-6A has been demonstrated in oligodendrocytes [39].

HHV-6 latency was first observed in patient peripheral blood mononuclear cells (PBMCs) and described as a covalent linkage between HHV-6 DNA and high molecular weight cellular DNA [40]. This was a curious finding considering that at the time all human herpesviruses were known to establish latency by forming an extrachromosomal episome. Years later, Arbuckle et al. demonstrated that both HHV-6A and HHV-6B predominately establish latency by integration into the telomeres of the host chromosome, and no circular episomes could be detected during latency [36,37]. HHV-6 integration is not site-specific and can potentially occur in the telomere region of any host chromosome of any HHV-6 infected cell. In vitro integration has been shown in a variety of cell lines including JJHan and Molt3 T-cells and the human embryonic kidney cell line HEK293T [36,37].

Chromosomally integrated HHV-6 has been reported both in vivo and in vitro, and integration into gametes can result in the inheritance of HHV-6 [36,41,42,43]. This condition, commonly known as inherited chromosomally integrated HHV-6 (iciHHV-6), occurs in approximately 1% of the human population worldwide and is considered the major mode of congenital HHV-6 transmission [42,44]. The inherited viral genome is passed on to subsequent generations in a Mendelian manner, and all iciHHV-6-positive individuals harbor one copy of the viral genome in every nucleated cell. As a result, these individuals exhibit a persistent high viral load (1 × 10^6^–1 × 10^7^ copies/mL) in whole blood, and hair follicles, leukocytes, and other clinical samples are also positive [42,45,46,47,48]. 

Although unusual, it is important to note that there has recently been a single report that describes the integration of HHV-6 in non-telomeric regions of the chromosome [49]. Sequencing of the integration site and fluorescent in situ hybridization confirmed integration events outside of the telomeres in four independent cell lines and at least one iciHHV-6 individual. The mechanism leading to these unusual integration events is likely distinct from homologous recombination, which is believed to result in typical HHV-6 integration, and may be a consequence of chromosomal instability resulting from the original integration event. 

## 6. Structure and Orientation of the Telomere-Integrated Viral Genome

**Figure 2:**
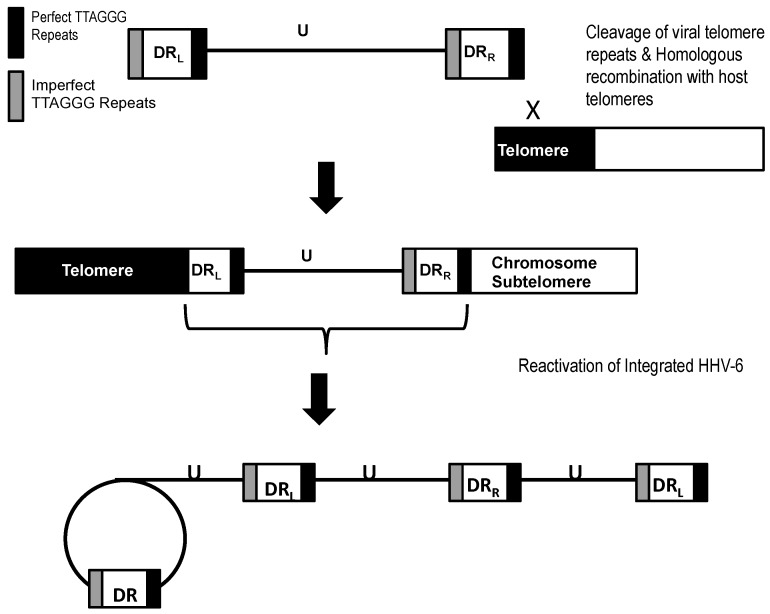
Model for integration and reactivation of HHV-6. The linear genome of HHV-6 integrates into the telomeres of the host chromosome, and during reactivation the integrated genome is liberated from the host chromosome, forming a circular intermediate. Rolling circle replication of the circular intermediate generates concatemers of the viral genome.

Most studies report that during in vitro and in vivo integration of HHV-6 a single copy of the viral genome is inserted into the subtelomere of the human chromosome (Figure 2). The viral genome is oriented so that the perfect telomere repeats at the right end of the viral genome adjoin the subtelomeres, and the imperfect telomere repeats at the left end of the viral genome adjoins the human telomeres [36,37,50]. In vitro experiments show integration of both, but not the entire left and right, direct repeats in a single copy of the viral genome. There have been reports that in some rare instances the number of direct repeats present during integration may vary in samples isolated from iciHHV-6 individuals. Multiplex ligation-dependent probe amplification (MLPA) analyses of five iciHHV-6B cases indicated direct repeats (DR) copy number greater than or less than two [50]. Similarly, when the DR copy number was assessed using quantitative polymerase chain reaction (qPCR) analysis, a DR copy number of greater than two was reported for numerous individuals with either iciHHV-6A or iciHHV-6B [49]. Furthermore, the DR copy number was reported to fluctuate in iciHHV-6 individuals over a span of three years. It is plausible that all or a portion of the HHV-6 genome can be lost if the ends of the telomeres are not protected. However, the mechanism by which DR duplication would occur has yet to be investigated. One possible explanation for the DR duplication is superinfection of iciHHV-6 positive individuals with an exogenous HHV-6 strain. 

**Figure 3:**
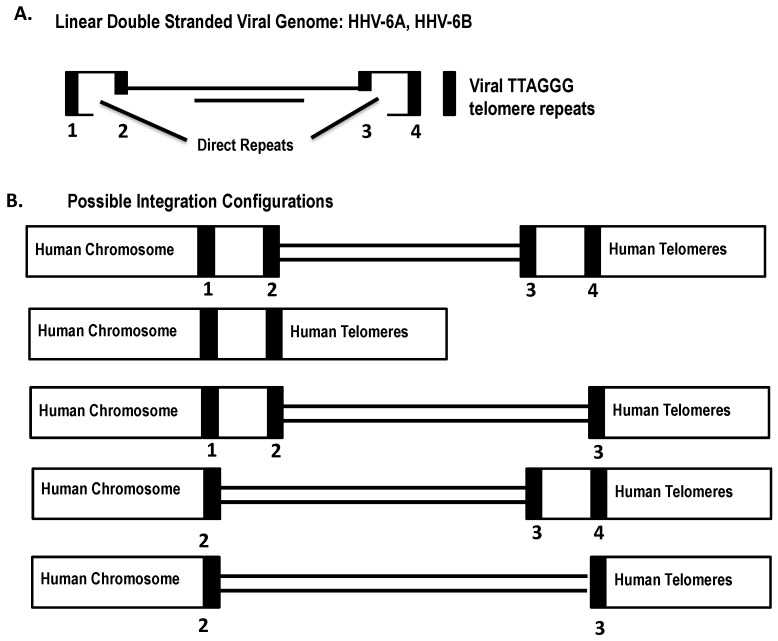
Possible integration configurations of the HHV-6 genome. (**A**) The HHV-6 genome contains four telomere-like repeats flanking the direct repeats; (**B**) Integration of the HHV-6 genome by recombination with the host telomeres can potentially occur at either of the four telomere repeats and five possible integration configurations may be generated.

Previous studies have analyzed the iciHHV-6 genome by various mapping approaches [36,37]. In all cases of HHV-6A and HHV-6B integration, a single copy of the viral genome was inserted in the human telomeres (Figure 2). In light of current knowledge of HHV-6, we hypothesize that several iciHHV-6 genome configurations may occur during integration (Figure 3). We propose that homologous recombination between the host telomeres and the viral telomere sequences can occur at either the perfect or imperfect telomere repeats, and in some cases a single direct repeat may be present or the direct repeats may be absent altogether.

## 7. Role of HHV-6 Telomeres and U94 in Integration

Human herpesvirus-6 telomere repeats have a number of important functions for integration and maintenance of the viral genome, but are not required for replication [50,51]. Viral telomere repeats are believed to trigger homologous recombination between host and viral telomere sequences. When the U2OS human osteosarcoma cell line, which allows the highest rate of integration, was infected with an HHV-6A strain lacking all telomeric repeats no integration events were evident [51,52]. Interestingly, the virus devoid of telomere repeats replicated similarly to the wild type virus in the JJHan T-cell line. The perfect telomere repeats, are the main determinants for both integration and genome maintenance [51]. When only the imperfect telomere repeats were present, integration events were negligible, but an HHV-6A strain containing only the perfect telomere repeats had an integration rate only slightly less than that of the wild type virus. The telomere repeats are believed to also play a role in genome maintenance. After integration, the imperfect telomere repeats likely serve as a template for telomere elongation in order to prevent senescence, and in the absence of either the perfect or imperfect telomere repeats genomic maintenance after infection is decreased [50,51].

The highly conserved HHV-6A/6B protein U94 is a putative integrase that could facilitate homologous recombination. U94, which is expressed during HHV-6 latency, is a homologue to the Rep78/68 integrase of the adeno-associated virus type 2 (AAV-2) and the two are believed to perform a biologically similar function [53,54,55]. The two proteins share only a 24% amino acid identity, but HHV-6 U94 has all the functional domains of Rep78/68 [36,53,55]. Rep78/68 possesses DNA binding, endonuclease, and helicase activity, which are essential for AAV-2 integration into a region outside of the telomere of the human chromosome 19. Similarly, U94 also displays the characteristics that would be necessary for HHV-6 integration by homologous recombination. HHV-6 U94 can non-specifically bind both single-stranded and double-stranded DNA but has a higher affinity for single stranded telomere-like sequences [53,54]. Additionally, U94 also has helicase, 3′–5′ exonuclease, and ATPase. Together, these functions should facilitate efficient integration of HHV-6 into the telomeres. 

Despite displaying all the properties of a functional integrase, recent studies suggest that U94 is not required for the integration of HHV-6 [56]. A recombinant HHV-6A virus lacking the U94 open reading frame integrated into the telomere of U2OS cells at a frequency similar to that of the wild type virus and its corresponding revertant mutant. There were also no notable differences in the proportion of nuclei that contained the viral genome and there was no effect on the maintenance of the viral genome in the infected cells. U94 was also shown to be nonessential in two other cell lines, HEK293T and JJHan. It is noteworthy that these studies are conducted in vitro using immortalized or cancer-derived cell lines; immortalized cells may express recombinases absent during natural infection. It is possible that U94 is involved in the integration or reactivation process during natural human infection. Additional studies are needed to uncover what cellular or viral factors drive the integration of HHV-6.

## 8. In Vitro Reactivation of Integrated HHV-6 from Infected Cells Harboring Latent HHV-6

Previous studies by Arbuckle et al. report in vitro reactivation of integrated HHV-6A triggered by histone deacetylase (HDAC) inhibitor trichostatin A (TSA) [36,37]. Upon treating latently infected HEK293T with varying amounts of TSA, HHV-6A U1102 was reactivated as evidenced by the detection of a circular replication intermediate by PCR amplification [37]. Rolling circle amplification and concatemers of the viral genome during lytic replication were confirmed by PCR amplification across a single direct repeat of the reactivated HHV-6. These data show that integration of HHV-6A in telomeres is not a dead-end phenomenon but is a reversible process, thus a novel mechanism of latency. Interestingly, when other independent HEK293T/HHV-6A cell lines were subject to the same TSA treatment reactivation did not occur. This suggests that in some cases HHV-6 integration is not reversible due to integration of a defective virus or that the specific site of integration may prevent reactivation. 

## 9. Superinfection of iciHHV-6 Individuals with a Second Virus and Reactivation of iciHHV-6

Early attempts to isolate infectious virus from peripheral blood isolated from individuals with iciHHV-6 failed, suggesting that inherited HHV-6 is completely inactive [41]. Analysis of freshly isolated PBMC from iciHHV-6 individuals using Gardella et al. vertical agarose gel electrophoresis that can detect as little as one copy/cell of viral DNA found no evidence of linear replicating viral DNA [36,57]. More recent attempts to confirm reactivation of an iciHHV-6 has focused on attempting to reactivate the latent virus by culturing cells harboring the integrated virus with naïve T-cells and reactivating the virus using epigenetic modulators. The emerging virus is then isolated from the freshly infected cells and individual genes sequences of the reactivated virus are compared to the known sequence of the inherited virus [36,37]. However, using this methodology has not yielded a conclusive result on whether or not iciHHV-6 reactivation always occurs.

In a unique group of iciHHV-6 patients suffering from neurological disorders viral late mRNA was detected using nested reverse transcriptase polymerase chain reaction (RT-PCR) [58]. Surprisingly, sequence analysis of the inherited viral genome and the mRNA showed significant mismatches suggesting that these patients harbor low level persistently replicating HHV-6. Interestingly the mRNA was no longer detectable after treatment with antiviral drugs suggesting direct involvement of a persistent secondary superinfection in the associated pathogenesis.

There have been more attempts and varying approaches to evaluate whether iciHHV-6 can be induced to produce reactivated virus using chemical agents known to reactivate latent herpesviruses. One approach was to expose T-cells isolated from iciHHV-6 patients to TSA or tetradecanoyl phorbol acetate (TPA). Reactivation was monitored by either PCR or co-cultivation with Molt-3 T-cells. HDAC inhibitor TSA treatment resulted in increase of HHV-6A copies per cell, and the Molt-3 cells showed signs of being infected by a reactivated virus (supplemental figures of [36]). However, the nucleotide sequence of the U94 gene of the virus isolated from Molt-3 cells closely resembled but was not identical to the inherited virus (supplemental figures of [36]). These results support the hypothesis that some iciHHV-6 patients may acquire a second HHV-6 strain after birth and that this second superinfecting strain is capable of persisting and could be involved in the pathogenesis of a neurological disease as described. A possible underlying immune tolerance to iciHHV-6 could explain these findings.

Despite evidence that iciHHV-6 individuals may be superinfected with a second HHV-6 strain, reactivation of the inherited strain cannot be excluded. In a recent study, sequencing of three iciHHV-6 European cardiac patients indicates that the inherited virus was intact and capable of reactivation [59]. Gravel et al. also reported the transplacental transmission of a reactivated iciHHV-6 strain from mother to child, in two separate instances and in the absence of inheritance [33]. Both mothers presented with iciHHV-6 and gave birth to children that were HHV-6 positive, but had not inherited the disease. Glycoprotein B sequences from the inherited virus and the virus isolated from the infant cord blood samples contained polymorphisms that were unique to each mother and distinct from other known HHV-6 isolates. The contradictions between the mentioned studies suggest that the ability of iciHHV-6 viruses to reactivate is a complex process, and may vary from one individual to another. Immune status of iciHHV-6 positive individuals may help to determine if reactivation will occur. Furthermore, acquired nucleotide changes to the integrated virus sequences over time may result in a dead-end virus that is incapable of reactivation. 

## 10. Hypomethylation of Subtelomere Regions, HHV-6B Integration and Pathogenesis

A recent study was conducted to evaluate epigenetic modifications of chromosomal DNA shortly after HHV-6B infection [60]. Subtelomere DNA of approximately 1 Mbp of Molt-3 T-cell line chromosomes was found hypomethylated. Interestingly the end of chromosome 17p13.3, an HHV-6 integration site reported in previous studies, had higher level of hypomethylation than other subtelomeres. Further experiments are required to determine whether integration is a trigger for hypomethylation of sequences adjacent to the integrated viral genome. If this scenario is correct, integration may induce subtelomere-specific genes leading to unique and possibly pathological effects. HHV-6 could theoretically integrate in either end of the 23 human chromosomes, and hypomethylation may cause 46 unique changes in gene expression. Alternatively, although we believe it is less likely, HHV-6B might specifically induce “generalized” subtelomere-specific hypomethylation. Regardless of the underlying mechanism, future studies are necessary to determine if hypomethylated subtelomere gene expression may be linked with pathological consequences.

## 11. Future of the Field of HHV-6

Very little information is currently available about the genetic variability of inherited HHV-6 viruses, and future studies should focus on the sequence analysis of inherited HHV-6 viruses in detail. Thus far, only one iciHHV-6A strain has been fully sequenced [59]. The nucleotide and amino acid sequences of inherited HHV-6 viruses most likely differ from each other and other previously characterized HHV-6 viruses. Full genome sequencing capabilities have increased greatly and should be applied to this topic. Whole-genome sequencing of inherited HHV-6 viruses may reveal critical antigen epitope variability in addition to mutations with the potential to cause viral replication defects and determine whether inherited viral genome expression occurs. Furthermore, it is also possible that some HHV-6 viruses more readily infect gametes than others. Differences in tropism may be one reason why iciHHV-6 is present in only a small fraction of the population, although HHV-6 viruses are ubiquitous.

To date, HHV-6 integration has been identified in the telomere of the X chromosome and 11 of the autosomal chromosomes (1, 6, 7, 9, 10, 11, 12, 17, 18, 19, 22) [36,40,41,43,46,59,61,62,63,64,65,66,67,68,69,70,71,72,73,74,75]. However, no studies indicate that HHV-6 integration is chromosome specific. In a cohort of European iciHHV-6 patients, 17p integration was predominant and the authors suggest that 17p integration was the first integration site and all others arose due to recombination in more recent evolutionary ancestors [59]. We offer an alternative hypothesis that the limited variation in integration sites suggests that HHV-6 integration during development of the fetus was not maintained because integration site was detrimental to the health of the host. Recently, iciHHV-6 was identified as a risk factor for the development of angina pectoris and acute graft-versus-host disease (GVHD) in hematopoietic cell transplant donor-recipient pairs [76,77]. These groundbreaking studies on the disease associations with iciHHV-6 are only the foundation and should pave the way for future studies that identify disease associations with specific integration sites, possible aberrant expression of subtelomere-encoded genes due to hypomethylation and possible other *cis*-acting effects of the integrated viral DNA. It would be interesting to explore association between specific HHV-6 integration sites and certain diseases. Further studies on immune tolerance due to expression of HHV-6 proteins are also warranted. This type of analysis may present a clearer picture of how iciHHV-6 contributes to illnesses.
